# Detection and Characterization of Extracellular Vesicles in Sputum Samples of COPD Patients

**DOI:** 10.3390/jpm14080820

**Published:** 2024-08-01

**Authors:** Ourania S. Kotsiou, Katerina Katsanaki, Aikaterini Tsiggene, Sophia Papathanasiou, Erasmia Rouka, Dionysios Antonopoulos, Irene Gerogianni, Nikolaos A. A. Balatsos, Konstantinos I. Gourgoulianis, Irene Tsilioni

**Affiliations:** 1 Laboratory of Human Pathophysiology, Faculty of Nursing, University of Thessaly, 415 00 Larissa, Greece; 2 Department of Biochemistry, University of Thessaly, 415 00 Larissa, Greece; kakats1992@yahoo.gr (K.K.); ktsiggene@gmail.com (A.T.); diadonop@uth.gr (D.A.); balatsos@bio.uth.gr (N.A.A.B.); 3 Department of Respiratory Medicine, University of Thessaly, 415 00 Larissa, Greece; papathanasiousophia@gmail.com (S.P.); igerogianni@yahoo.gr (I.G.); kgourg@med.uth.gr (K.I.G.); 4 Faculty of Nursing, University of Thessaly, 415 00 Larissa, Greece; errouka@uth.gr; 5 Department of Immunology, Tufts University School of Medicine, Boston, MA 02111, USA

**Keywords:** biomarkers, COPD, exosomes, extracellular vesicles, sputum, lung

## Abstract

Background: Only one study has reported the presence of extracellular vesicles (EVs) in COPD patients’ sputum. Thus, we aimed to isolate and characterize EVs from COPD and healthy individuals’ sputum. Methods: A total of 20 spontaneous sputum samples from COPD patients (m/f: 19/1) and induced sputum samples from healthy controls (m/f: 8/2) were used for EV isolation. The sputum supernatants were resuspended in PBS, precleared by centrifugation at 800× *g* for 10 min at 4 °C, and passed through a 0.22 μm filter (Millipore, Burlington, MA, USA). EVs were isolated by a standard membrane affinity spin column method (exoEasy maxi kit, Qiagen, Hilden, Germany). The EVs were then characterized by assessing their morphology and size using Transmission Electron Microscopy (TEM) and determining the CD9 and CD81 EV-markers with Western blot analysis. Results: The EVs had a spherical shape and their mean diameter in the COPD patients was significantly greater than in the controls. Enrichment of the EV markers, CD9 and CD81, were detected in both the healthy and COPD individuals. Total EV-associated protein was significantly increased in the COPD patients compared to the controls. ROC analysis showed that total EV-associated protein in the sputum could be used to differentiate between the controls and COPD patients, with a sensitivity of 80% and a specificity of 70% at a cut-off point of 55.59 μg/mL (AUC = 0.8150). Conclusions: EVs were detectable in both the COPD and healthy individuals’ sputum. The ratio of EVs in the 150–200 nm range was twice as high in the COPD patients than in the controls. The COPD patients’ sputum contained increased total EV-associated protein as compared to controls, highlighting their value as a new source of specific exoproteins.

## 1. Introduction

Chronic Obstructive Pulmonary Disease (COPD) remains a public health concern globally, characterized by persistent respiratory symptoms and airflow limitation due to airway and/or alveolar abnormalities [1]. Despite significant advances in understanding and treating COPD, it continues to be a leading cause of morbidity and mortality worldwide [1]. The complexity of COPD pathogenesis, which involves chronic inflammation, airway remodeling, and systemic effects, necessitates ongoing research into innovative diagnostic and therapeutic strategies.

Extracellular vesicles (EVs) have emerged as important players in the cellular landscape, mediating a plethora of physiological and pathological processes [2]. These small, membrane-bound particles are released by cells into the extracellular environment and are involved in a wide range of biological processes, including intercellular communication and disease propagation [3]. EVs encompass a variety of subtypes and size ranges, including exosomes, microvesicles, and apoptotic bodies, each characterized by distinct biogenesis pathways and molecular compositions. The contents of these EVs, such as proteins, lipids, and nucleic acids (including mRNA and miRNA), vary significantly depending on their cellular origin and the physiological or pathological conditions under which they are produced. These molecular constituents enable EVs to significantly influence recipient cells [4]. This intercellular transfer of molecular information via EVs is critical in many diseases, including cancer, neurodegenerative diseases, and inflammatory conditions [5,6].

In respiratory diseases, particularly COPD, EVs have been shown to mediate responses to environmental stressors, such as cigarette smoke and pollutants—key factors in the development and exacerbation of the disease [7]. The ability of EVs to transport bioactive molecules suggests that they could play crucial roles in the modulation of inflammatory responses, tissue repair processes, and pathogen responses within the pulmonary environment [8].

The study of EVs in COPD, however, is still in its infancy. To date, only one study has reported the presence of EVs in the sputum of COPD patients, underscoring a significant gap in the current research landscape [9]. This gap presents a unique opportunity to explore the utility of sputum-derived EVs as novel biomarkers for COPD. Biomarkers that can be easily and non-invasively obtained, such as those from sputum, are ideal for monitoring disease progression and response to therapy in COPD patients. Furthermore, given the accessibility of sputum samples, they represent a promising avenue for routine clinical assessments and longitudinal studies.

In addition, the dynamic range of molecules encapsulated within EVs can provide a snapshot of the cellular and molecular mechanisms active during disease states. The identification and characterization of these biomolecules within EVs could lead to the discovery of novel diagnostic markers and therapeutic targets, offering the potential for significant advancements in the management of COPD. Understanding the molecular signatures of EVs from COPD patients compared to healthy individuals can reveal insights into the pathophysiological processes underpinning COPD and potentially guide the development of tailored therapeutic intervention.

The objective of this study was to isolate and characterize extracellular vesicles from the sputum of both the COPD patients and healthy individuals.

## 2. Materials and Methods

### 2.1. Sample Collection

For this study, 20 spontaneous sputum samples were collected from COPD patients (19 males, 1 female) who had been diagnosed according to the GOLD (Global Initiative for Chronic Obstructive Lung Disease) criteria. Additionally, 10 induced sputum samples were collected from the healthy controls (8 males, 2 females) to serve as comparative non-disease controls. The collection was performed at the Department of Respiratory Medicine, University of Thessaly, Greece, following ethical guidelines and with all the participants providing informed consent. Samples were processed within 2 h of collection to minimize degradation of the cellular material.

### 2.2. Isolation of Sputum Extracellular Vesicles

EVs were isolated using a standard membrane affinity spin column method, specifically the exoEasy Maxi Kit (Qiagen, Valencia, CA, USA), from 1 mL of sputum as previously described [10,11]. This method allowed for the efficient and reproducible isolation of exosomes from various biological fluids. The sputum samples were initially treated with dithiothreitol to reduce mucosity, centrifuged at 800× *g* for 10 min at 4 °C to remove cells and debris, and filtered through a 0.22 μm pore filter to ensure the removal of larger particles. Pre-filtered sputum was then mixed with buffer XBP and bound to an exoEasy membrane affinity spin column. The bound extracellular vesicles were washed with Buffer XWP, eluted with 250 μL Buffer XE (an aqueous buffer containing primarily inorganic salts), and were then ready to use for further analysis.

### 2.3. Transmission Electron Microscopy

The isolated EVs were characterized for size and morphology using Transmission Electron Microscopy (TEM) at Harvard Medical School’s Electron Microscopy (EM) Core Facility. Samples for TEM were prepared by placing a drop (5 μL) of isolated sputum-derived EVs suspended in Buffer XE onto formvar-carbon-coated grids for 1 min. The grids were then negatively stained by incubating with 2 µL of uranyl formate 0.75% for 30 s and were scanned with a TecnaiG2 Spirit BioTWIN TEM (FEI, Hillsboro, OR, USA).

### 2.4. Protein Quantification and Western Blot Analysis

The presence of specific EV markers, CD9 and CD81, was confirmed by Western blot analysis, conducted at Tufts University to validate the isolation procedure, and ensure the purity of the vesicle preparations. The concentration of total protein was quantified by the bicinchoninic acid (BCA) assay (Thermo Fisher Inc., Rockford, IL, USA), using bovine serum albumin (BSA) as standard. Protein samples of 10 μg were loaded per lane and separated on 4–12% NuPAGE Bis-Tris gels under SDS-denaturing conditions (Invitrogen Life Technologies, Grand Island, NY, USA), starting with 65 V for 45 min and then increasing to 90 V for another 30 min. Proteins were then electrotransferred onto nitrocellulose membranes (Bio-Rad, Hercules, CA, USA), followed by blocking for 1 h using 5% BSA in Tris-buffered saline containing 0.05% Tween-20. The membranes were then incubated overnight at 4 °C with the following primary antibodies at 1:1000 dilutions: CD9 (EXOAB-CD9A-1), CD81 (EXOAB-CD81A-1) (System Biosciences, Mountain View, CA, USA), and calnexin (cat#sc-23954, Santa Cruz Biotech, Dallas, TX, USA) [12]. Finally, the membranes were incubated with the appropriate secondary horseradish peroxidase (HRP)-conjugated antibody (System Biosciences) at 1:20,000 dilutions for 1 h at room temperature, and the blots were visualized by enhanced Super Signal West Pico Chemiluminescence (Fisher Scientific, Pittsburgh, PA, USA).

### 2.5. Statistical Analysis

The concentration of total EV-associated protein was compared between the healthy controls and COPD samples using the Mann–Whitney U non-parametric test following the examination of normality of distribution using the Shapiro-Wilk test. Results are presented as mean ± SD. The significance of comparisons is denoted by *p* < 0.05. Receiver operator characteristics (ROC) analysis was constructed and the area under the curve (AUC) was calculated in order to evaluate the diagnostic performance of sputum-derived EV total protein in differentiating between healthy controls and COPD patients. The optimum cut-off point was established from the ROC analysis by selecting the value that provided the greatest sum of sensitivity and specificity, i.e., the point closest to the upper left corner of the ROC plot. For the optimum cut-off point provided by each ROC analysis, sensitivity and specificity were calculated using standard formulas. All analyses were performed using GraphPad Prism 5 software (GraphPad Software, San Diego, CA, USA).

## 3. Results

### 3.1. Characterization of Sputum-Derived EVs from COPD Patients

**Figure 1:**
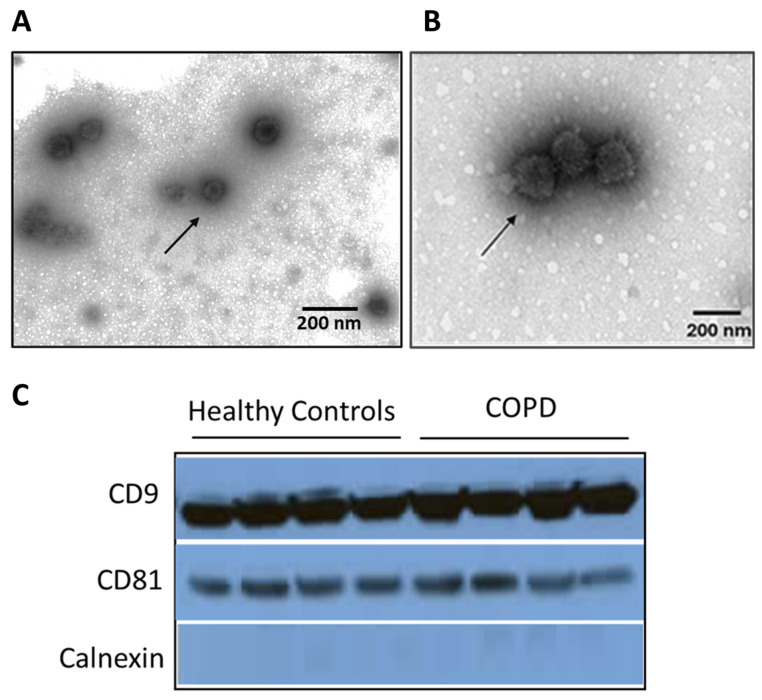
Transmission electron microscopic image of sputum-derived extracellular vesicles from (**A**) Healthy Control and (**B**) COPD patient. Extracellular vesicles (EVs) with round shape morphology with up to 200 nm size are present (left and right, bar: 200 nm). (**C**) Western blot analysis of exosomes collected from sputum samples of healthy controls (HC) and COPD patients. The presence of the exosome specific tetraspanins, CD81 and CD9, as well as the non-exosomal protein marker calnexin was assessed.

Transmission Electron Microscopy (TEM) was employed to characterize the morphology and size distribution of EVs isolated from the sputum of 20 COPD patients and 10 healthy controls (Figure 1A,B). The characteristics of the COPD patients are presented in Table 1. The analysis confirmed the vesicular nature of the isolated particles, with the majority displaying a rounded, bilipid-layered structure characteristic of EVs. Notably, there was a distinct pattern in size distribution between the two groups. EVs from COPD patients exhibited a prevalence of vesicles predominantly within the 150–200 nm range, which comprised approximately 60% of the observed population. In contrast, healthy controls showed a more even distribution across the size spectrum, with only about 30% of the vesicles falling into the 150–200 nm range. Quantitative analysis of the images from 30 randomly selected fields for each sample type revealed that the mean diameter of EVs from the COPD patients was significantly greater than that of healthy controls (mean ± SD: 145 ± 25 nm vs. 115 ± 20 nm; *p* < 0.001). This suggests a potential alteration in EV biogenesis or selective release mechanisms in the pathophysiology of COPD.

To confirm the exosomal nature of the isolated vesicles and investigate the potential quantitative differences between the groups, Western blot analysis was conducted for the common exosomal markers, CD9 and CD81. Calnexin was used as a negative control. Both CD9 and CD81 markers were detectable in all samples, validating the exosomal isolation method (Figure 1C). However, there were no apparent differences in the expression of CD9 and CD81 in the COPD patient group compared to the healthy controls.

### 3.2. Assessment and Diagnostic Accuracy of Sputum EV-Associated Total Protein

**Figure 2:**
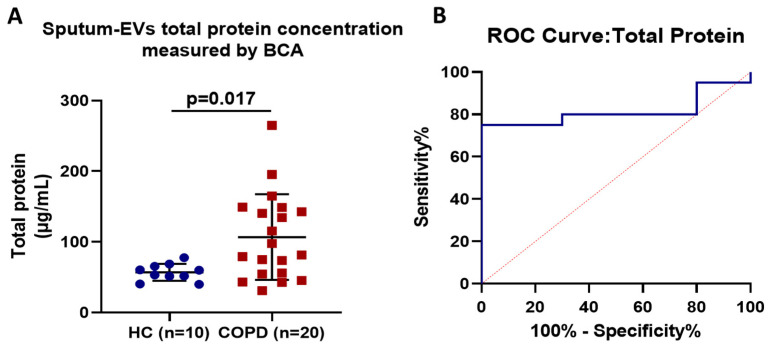
Scattergrams present EV-associated total protein in the sputum of COPD patients compared to healthy controls. (**A**) Total EV-associated protein isolated from sputum samples of COPD patients (*n* = 20) compared to healthy controls (*n* = 10). (**B**) Diagnostic performance of sputum EV-associated total protein according to ROC Analysis. Sputum EV-associated protein was an accurate to test to differentiate between COPD and healthy individuals with a sensitivity of 80% and a specificity of 70% at a cut-off point of 55.59 μg/mL (AUC = 0.8150).

Sputum EV-associated protein was increased in patients with COPD (Figure 2). The total protein concentration in EV-enriched sputum samples was significantly higher in COPD patients (106.7 ± 60.56 μg/mL) as compared to the healthy controls (56.82 ± 11.99 μg/mL), *p* = 0.017. According to ROC analysis, sputum-derived EV-associated protein was found to be an accurate test to differentiate between healthy controls and COPD patients, with a sensitivity of 80% and a specificity of 70% at a cut-off point of 55.59 μg/mL (AUC = 0.8150).

## 4. Discussion

This study provides important insights into the characteristics of EVs in the sputum of COPD patients compared to healthy controls. Our findings indicate significant differences in EV morphology and protein content, which are likely reflective of the underlying disease mechanisms in COPD.

EVs are heterogeneous in nature, encompassing a variety of subtypes including exosomes, microvesicles, and apoptotic bodies. The size and subtype of EVs can influence their content and functional roles in intercellular communication. Each of these subtypes is characterized by distinct biogenesis pathways and molecular compositions, contributing to their functional diversity. Exosomes, typically ranging from 30 to 150 nm in diameter, originate from the endosomal pathway. Microvesicles, which are larger (100 to 1000 nm), bud directly from the plasma membrane, while apoptotic bodies are the largest (500 to 2000 nm) and are released during programmed cell death.

Here, we found that 60% of EVs from the COPD patients were in the 150–200 nm size range, while in the healthy individuals, only 30% of EVs fell within this range. This means the ratio of EVs in the 150–200 nm range was twice as high in the COPD patients compared to the healthy individuals. The observed increase in the size of EVs in COPD patients might reflect an altered state of cellular stress or activation in the respiratory tract [4,13,14,15,16,17]. Larger EVs have been associated with cellular stress responses in various diseases and could indicate an adaptive response to the chronic inflammatory environment in COPD [4,13,14,15,16,17]. This is supported by the literature suggesting that EVs are involved in the modulation of inflammatory processes and can act as vehicles for the propagation of inflammatory signals across cell membranes [4,13,14,15,16]. The size of EVs might influence their ability to encapsulate and transport bioactive molecules, including proteins, which can interact with recipient cells and influence disease pathology [13,14,15,16].

Exosomes contain various types of proteins on their surface, such as CD9, CD63, CD81, HSP70, TSG101, and ALIX, which can be used to identify them [18,19,20]. In our study, both CD9 and CD81 were detectable in all samples; however, there were no apparent differences in their expression between COPD and healthy controls.

Moreover, exosomes contain a variety of internal markers, such as lipids, proteins and nucleic acids (including mRNA and miRNA) and thus can have effects to multiple target cells within a signaling pathway [18,19,20]. Therefore, the protein content can be used to determine the amount of exosomes isolated from the media.

Here, we showed that the sputum-derived total EV-associated protein levels were significantly increased in patients with COPD compared to the healthy controls. This could be potentially due to higher protein cargo enclosed in many exosomes in COPD patients, suggesting an upregulation of protein packaging mechanisms in response to the disease environment. This finding aligns with previous studies that have reported increased levels of EVs in the plasma and sputum of COPD patients, highlighting their potential role in disease progression [21,22,23,24,25,26]. Genschmer et al. demonstrated that lung-derived EVs were more abundant in the sputum of COPD patients and contained higher levels of pro-inflammatory proteins, which contributed to the disease pathology [26], while Takahashi et al. found that EVs from COPD patients exhibited altered protein composition, including increased levels of proteases and inflammatory mediators. This suggests that the disease environment in COPD drives changes in EV biogenesis and cargo loading [22]. The literature further indicates that specific protein signatures within EVs can differentiate between COPD patients and healthy controls [27].

However, the cellular origin of the increased sputum EVs is presently unknown. Fujita et al. [28] showed that EVs from COPD patients can induce the release of inflammatory cytokines from bronchial epithelial cells. Macrophages and neutrophils have been reported to release EVs containing various proteins (e.g., cytokines and matrix metalloproteinases) and promoting inflammation of COPD [25]. Another example is the release of exosomes from activated neutrophils that contain surface-bound neutrophil elastases, which are resistant to degradation by α1-antitrypsin, leading to excessive alveolar destruction [26].

The pronounced differences in EV characteristics between the COPD patients and the healthy controls underscore the potential utility of EVs as biomarkers for COPD. The ability to detect and quantify specific EV attributed non-invasively from sputum samples could transform the current diagnostic and monitoring paradigms for COPD. Concerning the vital roles of proteins within exosomes, proteomic analyses of whole exosomes should be performed to screen biomarkers for COPD, while the combined use of EV size and protein markers could offer a promising approach for early detection and severity assessment of COPD.

Moreover, the current literature indicates that EVs play significant roles in intercellular communication and are involved in the pathogenesis of both COPD and lung cancer. Studies have shown that the size of EVs can vary depending on the disease state. For instance, COPD patients exhibit larger EVs compared to healthy controls, as observed in our study. However, distinguishing pre-cancerous or non-cancerous states in COPD patients purely based on EV size may be challenging. The literature suggests that while EV size can indicate cellular stress or activation, it is not specific enough to differentiate between COPD and lung cancer, as both conditions can lead to the production of larger EVs due to increased cellular turnover and inflammation [29,30].

Protein cargo within EVs offers more specific insights into disease states. COPD patients have been reported to have EVs with higher protein content, which includes inflammatory cytokines, proteases, and other molecules involved in tissue remodeling and immune responses [31]. In the context of lung cancer, EVs also carry oncogenic proteins, growth factors, and other tumor-associated markers [32]. Therefore, while increased protein levels in EVs are common in both COPD and lung cancer, specific protein signatures associated with oncogenesis might help differentiate between pre-cancerous or non-cancerous COPD states.

Research has identified several protein biomarkers within EVs that could potentially differentiate between COPD and lung cancer. For instance, the presence of tumor-associated proteins such as EGFR, PD-L1, and TGF-β1 in EVs has been linked to lung cancer [4]. In contrast, EVs from COPD patients might predominantly carry proteins associated with chronic inflammation and tissue degradation, such as matrix metalloproteinases (MMPs) and various cytokines [33].

In other words, while the size of EVs alone may not be a definitive marker for distinguishing pre-cancerous or non-cancerous COPD patients, the protein content within these EVs offers a more promising avenue. By analyzing specific protein signatures and comparing the relative abundance of oncogenic versus inflammatory proteins, it may be possible to differentiate between COPD patients at risk of developing lung cancer and those who are not. A comprehensive approach that combines EV size analysis with detailed proteomic profiling is necessary to achieve reliable differentiation between pre-cancerous and non-cancerous COPD states. Future studies should focus on identifying and validating specific EV-associated biomarkers that can accurately predict the progression from COPD to lung cancer.

Our study, although promising, has several limitations that need to be acknowledged. The sample size, particularly of the healthy controls, was relatively small, which may affect the generalizability of the results. The Western blot analysis of EV-specific markers is missing a normalization protein as well as a positive control for calnexin expression. Additionally, the cross-sectional nature of the study limits our ability to infer causality or the directionality of the observed changes. Longitudinal studies involving larger cohorts are necessary to validate these findings and elucidate the dynamics of EV changes over the course of COPD progression and treatment. Measuring total protein levels in EVs provides valuable insights into their molecular composition and potential biomarker capabilities. However, comparative studies across different diseases are needed to establish diagnostic relevance. The existing literature supports the notion that EVs from various diseases, including lung cancer, carry distinct protein signatures that may reflect disease-specific pathophysiology [31,32]. These proteins can include oncogenic markers, growth factors, and inflammatory molecules, which are crucial for disease characterization and potentially for diagnostic purposes.

As a pilot study, our primary objective was to establish feasibility and gather preliminary data regarding EV characteristics in COPD patients. This preliminary exploration will guide larger-scale studies focused on clinical relevance. Moving forward, we envision expanding our analysis to include refined EV subtyping by implementing techniques such as nanoparticle tracking analysis (NTA) or flow cytometry to better characterize EV subtypes based on size, morphology, and surface markers in COPD patients compared to healthy controls. Moreover, future studies should aim to correlate EV characteristics with clinical outcomes and disease stages in COPD to better understand their potential as biomarkers and therapeutic targets. This will provide initial insights into disease-specific molecular signatures, as well as exploratory clinical insights by exploring potential correlations between EV-associated molecules and clinical parameters of COPD severity or progression in a cohort. Furthermore, future research should focus on assessing sputum-derived EV miRNA levels and elucidating the specific molecular pathways influenced by these miRNAs in COPD patients, as well as exploring the potential therapeutic implications of modulating these pathways.

## 5. Conclusions

This study successfully isolated and characterized EVs from the sputum of COPD patients and healthy individuals. EVs in both groups exhibited a spherical shape with sizes ranging from 50 to 200 nm; however, the observed increase in the size of EVs in COPD patients might reflect an altered state of cellular stress or activation in the respiratory tract, as the ratio of EVs in the 150–200 nm range was twice as high in COPD patients compared to healthy individuals. Notably, the protein cargo within EVs from COPD patients was markedly enriched, as the total EV-associated protein levels were significantly higher in COPD patients compared to healthy controls, indicating a potential alteration in the EV biogenesis or release mechanisms in COPD. The presence of EV markers CD9 and CD81 was also confirmed, while an absence of calnexin, a non-exosomal marker, was identified.

These findings, although preliminary, underscore the diagnostic potential of sputum EVs in COPD. Further studies should focus on miRNA profiling of sputum EVs which is expected to reveal distinct expression patterns linked to several biological pathways involved in immune regulation, cytokine signaling, apoptosis, etc. This highlights the value of sputum EV analysis in developing non-invasive diagnostic and therapeutic strategies for COPD.

## Figures and Tables

**Table 1:** COPD patients (20) and Healthy Controls (10) demographic and clinical characteristics.

Parameters	COPD Patients (*n* = 20)	Controls (*n* = 10)	*p*-Value
Gender			
Males, n (%)	19 (95)	9 (90)	0.569
Females, n (%)	1 (5)	1 (10)	
Age (years)	70 ± 8	65 ± 5	0.342
BMI (Kg/m^2^)	31 ± 6	28 ± 7	0.423
Commorbidities, yes, n (%)			
Atrial Hypertention	18 (90)	5 (50)	0.140
Hyperlipidemia	15 (70)	5 (50)	0.240
Obstructive sleep apnea	10 (50)	3 (30)	0.321
Depression	9 (45)	2 (20)	0.150
Pys	77 ± 42	8 ± 2	0.001
FEV1/FVC	66 ± 5	76 ± 2	0.001
FEV1 (%)	72 ± 8	110 ± 2	0.001
GOLD stage		-	-
Stage I, n (%)	10 (50)		
Stage II, n (%)	9 (45)		
Stage III, n (%)	1(5)		
Stage IV, n (%)	0 (0)		
CAT score	16 ± 9	-	-
Εxacerbation the last year, yes, n (%)	6 (30)	-	-
Hospitalization the last year, yes, n (%)	6 (30)	-	-

Abbreviations: BMI, body mass index; CAT, COPD Assessment Test; FEV1, Forced expiratory volume in the first second; FVC, Forced Vital Capacity.

## Data Availability

The data presented in this study are available on request from the corresponding author. The data are not publicly available due to privacy reasons.
